# Sigma-1 receptor targeting inhibits connexin 43 based intercellular communication in chronic neuropathic pain

**DOI:** 10.1007/s00011-024-01926-0

**Published:** 2024-08-02

**Authors:** Simona Denaro, Simona D’Aprile, Filippo Torrisi, Agata Zappalà, Agostino Marrazzo, Mahmoud Al-Khrasani, Lorella Pasquinucci, Nunzio Vicario, Rosalba Parenti, Carmela Parenti

**Affiliations:** 1 Section of Physiology, Department of Biomedical and Biotechnological Sciences, University of Catania, 95123 Catania, Italy; 2 Department of Medicine and Surgery, University of Enna “Kore”, 94100 Enna, Italy; 3 Section of Medicinal Chemistry, Department of Drug and Health Sciences, University of Catania, 95123 Catania, Italy; 4 Department of Pharmacology and Pharmacotherapy, Faculty of Medicine, Semmelweis University, Budapest, Hungary; 5 Section of Pharmacology and Toxicology, Department of Drug and Health Sciences, University of Catania, 95123 Catania, Italy

**Keywords:** Neuropathic pain, Connexin 43, Gap junction, Glial cell, Sigma-1 receptor

## Abstract

**Background and objective:**

Neuropathic pain is a chronic condition characterized by aberrant signaling within the somatosensory system, affecting millions of people worldwide with limited treatment options. Herein, we aim at investigating the potential of a sigma-1 receptor (σ1R) antagonist in managing neuropathic pain.

**Methods:**

A Chronic Constriction Injury (CCI) model was used to induce neuropathic pain. The potential of (+)-MR200 was evaluated following daily subcutaneous injections of the compound. Its mechanism of action was confirmed by administration of a well-known σ1R agonist, PRE084.

**Results:**

(+)-MR200 demonstrated efficacy in protecting neurons from damage and alleviating pain hypersensitivity in CCI model. Our results suggest that (+)-MR200 reduced the activation of astrocytes and microglia, cells known to contribute to the neuroinflammatory process, suggesting that (+)-MR200 may not only address pain symptoms but also tackle the underlying cellular mechanism involved. Furthermore, (+)-MR200 treatment normalized levels of the gap junction (GJ)-forming protein connexin 43 (Cx43), suggesting a reduction in harmful intercellular communication that could fuel the chronicity of pain.

**Conclusions:**

This approach could offer a neuroprotective strategy for managing neuropathic pain, addressing both pain symptoms and cellular processes driving the condition. Understanding the dynamics of σ1R expression and function in neuropathic pain is crucial for clinical intervention.

## Introduction

Neuropathic pain, a debilitating chronic disorder of the somatosensory nervous system, is estimated to affect 7–10% of the general population, leading to considerable morbidity, reduced quality of life and elevated healthcare costs [1, 2]. At the clinical level, symptoms of neuropathic pain include paresthesia, characterized by abnormal tingling, prickling and burning sensation, as well as thermal or mechanical hypersensitivity, which manifests as an exaggerated response to temperature or touch of physiologically non-noxious stimuli [3]. From a pathophysiological perspective, unravelling the intricate mechanisms underlying this complex condition is a critical step in developing effective therapeutic strategies. While initial research focused primarily on neuronal alterations in the peripheral nervous system (PNS) or central nervous system (CNS), recent advances have highlighted the pivotal role of non-neuronal cells in the chronicization process of the disease [4]. Neuroinflammation, characterized by activated glial cells and the release of pro-inflammatory mediators, plays a critical role in the development and maintenance of chronic pain [5, 6]. Microglia and astrocytes, historically considered as supportive elements of the CNS, are now reported to contribute to the pathogenesis of chronic pain through a cascade of events following the initial stage of neuronal injury [7, 8]. Reactive glial cells release pro-inflammatory mediators, leading to remodeling and dynamic changes in the CNS microenvironment, resulting in a vicious cycle sustaining pain hypersensitivity and amplifying excitotoxic and neurodegenerative mechanisms [9–11]. Gap junction (GJ) communication between glial cells becomes pivotal in understanding the maintenance of neuropathic pain [12]. Connexin 43 (Cx43), the predominant GJ-forming protein expressed by astrocytes, has been implicated in the pathogenesis of the neuropathy [13, 14]. Experimental studies exploring the role of GJs and Cxs have demonstrated their involvement in astrogliosis and microgliosis, amplifying and remodeling reactive responses to stimuli and insults. The upregulation of Cx43 following nerve injury and its subsequent effect on astrocyte-to-microglia cell coupling has been identified as a potential therapeutic target [15].

While anticonvulsants, antidepressants, and opioid analgesics are currently available as treatment options, their limited efficacy in the management of chronic pain, together with significant side effects, highlights the need to explore novel therapeutic approaches [16]. Initially identified as one of the subtypes of opioid receptors, sigma receptors (σRs) are now recognized as a unique family distinct from µ-, δ- and k-opioid receptors. The σ1R, first cloned in 1996 by Hanner and colleagues [17], is a chaperone protein predominantly expressed at the mitochondrial-endoplasmic reticulum membrane interface, with the ability to translocate to other subcellular organelles [18]. As such, it can interact with a plethora of targets, including voltage-gated ion channels, glutamate and γ-aminobutyric acid (GABA) ionotropic receptors, dopamine and acetylcholine receptors, neurotrophic tyrosine kinase receptor type 2 (TrkB), and intracellular targets such as kinases (e.g. Src kinase) and inositol triphosphate (IP3) [19, 20]. Consequently, σ1R has been implicated in a wide range of disorders, including amnesia, depression, stroke, Alzheimer’s disease, age-related cognitive impairment at the CNS level [21]. Moreover, due to its distribution in key area for pain control, such as dorsal root ganglia (DRG), spinal dorsal horn, periaqueductal grey matter, locus coeruleus, and rostroventral medulla, σ1R plays a prominent role in pain. Current preclinical evidence supports the modulatory role of the σ1R in nociception [22]. In contrast to opioid receptors, σ1R ligands do not modify physiological sensory mechanical and thermal thresholds. However, σ1R agonists counteract opioid receptor-mediated analgesia, acting as an endogenous anti-opioid system. Conversely, a marked enhancement of morphine-induced antinociception was observed with σ1R antagonists, both at central and peripheral levels [23]. In several models of pathological pain, including chronic constriction injury (CCI) of the sciatic nerve, cisplatin neuropathy, and formalin-induced inflammation, it has been reported that σ1R antagonists, when administered alone, have anti-hyperalgesic and anti-allodynic effects in conditions of heightened excitability due to sustained afferent input [24]. Using σ1R receptor knockout mice, it has been demonstrated that this receptor plays a crucial role in several models of pathological pain, including neuroinflammatory pain [25, 26]. In our previous studies, we demonstrated that σ1R inhibition by selective antagonists such as (−)-MRV3 and (+)-MR200 prevents thermal hyperalgesia, mechanical allodynia, and paw edema induced by intraperitoneal (i.p.) injection of carrageenan in a dose-dependent manner [27–29]. Importantly, σ1Rs are expressed on both microglia and astrocytes, modulating cellular processes by suppressing the release of pro-inflammatory mediators. This modulation influences microglial phagocytic activity, promoting debris clearance and tissue repair [30]. In this study, we aimed to evaluate (+)-MR200, a selective σ1R antagonist, in a model of neuropathic pain induced by CCI of the sciatic nerve and to uncover the cellular mechanisms involved in neuropathic pain chronicization. Our approach also sought to explore this σ1R antagonist as a therapeutic option to sustain neuroprotection and to reduce pro-inflammatory intercellular crosstalk between CNS resident cell populations.

## Materials and methods

### Dorsal root ganglion neurons derivation and culture

For DRG neurons derivation, the vertebral columns of a naïve male Sprague–Dawley rats were isolated and kept in dry iced Leibovitz's L-15 Medium (Gibco, Cat#11,415,064). Lumbar DRGs were extracted and placed in a petri dish in dry iced Leibovitz's L-15 Medium supplemented with 1 × pen/strep (Sigma-Aldrich, Cat#P4458). After, DRGs were collected and digested in collagenase (2 mg/mL) (Sigma-Aldrich, Cat#C9891) for 1 h at 37 °C, 5% CO_2_. Following enzymatic digestion, DRGs were mechanically triturated, loaded in 10 mL of 15% BSA (Sigma-Aldrich, Cat#A2058) in DMEM 4.5 g/L glucose (Gibco, Cat#11,966–025) in order to remove myelin and debris. Then DRGs were centrifugated at 1′000 g for 5 min. Cells were collected, suspended in DRG neuron medium of DMEM (4.5 g/L glucose), supplemented with nerve growth factor (NGF) 2 ng/mL (Sigma-Aldrich, Cat#N6009), 1% GlutaMax (Gibco, Cat#35,050,061), 1% Penicillin–Streptomycin solution (Gibco, Cat#15,140–122), 1% Fetal bovine serum (FBS) (Gibco, Cat#A4766801) and plated in 24-well plates, coated with Matrigel (Corning, Cat#356,234). The following day, half of the medium was replaced with fresh DRG neuron medium, and neurons were cultured for the following 7 days, until they reached an optimal growth density for the following experiment.

### In vitro experiment on excitotoxic damage

On the day of the experiment, culture medium was removed and fresh medium supplemented with 100 μM picrotoxin (PTX), was added to PTX-stimulated cell cultures for 1 h. Control cell cultures received fresh media with vehicle (PBS). After PBS washing, PTX-stimulated cells were treated with respective drug-containing media as follows: 1 μM (+)-MR200, 1 μM PRE084, and a combination of 1 μM PRE084 (added 1 h prior) and 1 μM (+)-MR200, dissolved in DMSO (Sigma-Aldrich, Cat#D2438). Control group and PTX-stimulated untreated cells received fresh medium added with vehicle (PBS + DMSO). Cells were cultured for 24 h, then were fixed in PFA 4% with sucrose 2% for 10 min at room temperature (RT) and washed in PBS for immunofluorescence analyses.

### Animals

All experiments were performed on male Sprague–Dawley rats, weighing 200–250 g, purchased from Envigo Laboratories, in accordance with the European Communities Council directive and Italian regulations (EEC Council 2010/63/EU and Italian D.Lgs.no. 26/2014). All efforts were made to replace, reduce and refine the use of laboratory animals. Rats were randomly assigned to different cages (n ≤ 3) and housed with standard diet and water. Before all surgery procedures, rats were randomly divided into four groups: Sham vehicle (n = 4), CCI vehicle (n = 4), CCI (+)-MR200 (n = 8), CCI PRE084 (n = 8), CCI PRE084 (+)-MR200 (n = 8).

### CCI model of neuropathic pain and drug administration

The CCI model used to induce neuropathic pain was performed following the protocols of Bennett and Xie [31], with some minor adaptations. In brief, animals were anesthetized via isoflurane inhalation (4% induction, 2% maintenance), and an incision was made below the hipbone, parallel to the left common sciatic nerve. The sciatic nerve was then exposed, and four ligatures (4/0 chromic silk, Ethicon) were tightly tied around the nerve, proximal to the nerve trifurcation, with approximately 1 mm spacing between ligatures. This process continued until a brief twitch was observed in the respective hind limb. For the sham surgery, the sciatic nerve was exposed without the application of any ligatures.

Starting from 9 days post-ligatures (dpl) up to 16 dpl, rats received a daily subcutaneous injections of either vehicle, (+)-MR200 (1 mg/kg) and/or PRE084 (32 mg/kg) injected about 30 min before (+)-MR200 administration.

### Von frey behavioral test

Von Frey behavioral test was executed to evaluate the development of mechanical allodynia in response to a series of calibrated von Frey filaments with bending forces ranging from 0.02 to 30 g. Briefly, rats were placed in an elevated plexiglass chamber with a wire mesh bottom and left for approximately 1 h to acclimatize before starting the behavioral test. Filaments of increasing bending forces were applied perpendicular to the hind paw of the animal from below. The paw withdrawal threshold was assessed using the 'up–down' method [32].

### Ex vivo tissue preparation

At 16 dpl, rats were anesthetized with an intraperitoneal injection of ketamine (10 mg/mL) and xylazine (1.17 mg/mL) and transcardially perfused with a solution of 0.5 M EDTA (Sigma) in normal saline, followed by ice-cold 4% paraformaldehyde (PFA) in PBS. Then, spinal cords were dissected out and post-fixed in 4% PFA overnight at + 4 °C. The day after, samples were washed in PBS and cryoprotected with a solution of 30% sucrose in PBS at + 4 °C for three days. Then, spinal cords were embedded in optimum cutting temperature (OCT) medium and rapidly frozen in liquid nitrogen, then stored at − 80 °C before proceeding to cryo-sectioning. 20 µm-thick sections were obtained and mounted on a microscope slide, then stored at − 80 °C.

### Immunofluorescence

For immunofluorescence staining on DRG derived neurons, cultures were washed in PBS and then incubated with 10% normal goat serum (NGS, Abcam, Cat#ab7481, RRID: AB_2716553) for 1 h at RT. Cells were then incubated overnight at + 4 °C with mouse monoclonal anti-class III β-tubulin (TUJ1) (BioLegend, Cat#801,201, RRID: AB_2313773, 1:200). On the following day, cells were washed with PBS three times for 5 min, then incubated 1 h at RT with the appropriate anti-mouse secondary antibody (Alexa Fluor 488, Thermo Fischer Scientific, Cat#A-11001, RRID: AB_2534069, 1:1000) [33].

For immunofluorescence staining on spinal cord, sections were washed in PBS, then incubated with NGS 10% (Abcam, Cat#ab7481, RRID: AB_2716553) or normal donkey serum (NDkS, Abcam, Cat#ab7475, RRID: AB_2885042) in PBS-0.3% Triton (Thermo Fisher Scientific, Cat#11,488,696, CAS: 9002–93-1) for 1 h at RT. Then, slides were incubated overnight at + 4 °C with the following primary antibodies, diluted in 1% NGS or 1% NDkS and PBS-0,3% Triton:, anti-mouse monoclonal anti-NeuN antibody (Merck Millipore, Cat#MAB377, RRID: AB_2298772, 1:100), goat anti-AIF/Iba1 antibody (Novus Biologicals, Cat#NB100-1028, RRID: AB_521594, 1:100), rabbit polyclonal anti-mouse monoclonal anti-glial fibrillary acidic protein (Gfap) antibody (Santa Cruz Biotechnology, Cat#610,566, RRID: AB_397916, 1:100), rabbit polyclonal anti-Cleaved Caspase-3 (Asp175) antibody (Cell Signaling Technology Cat#9661, RRID: AB_2341188, 1:300), rabbit polyclonal anti-Cx43 antibody (Cell Signaling Technology Cat#3512, RRID: AB_2294590, 1:100). The following day, after three times washing in PBS-0.3% Triton, samples were incubated for 1 h at RT, with the appropriate fluorescent secondary antibodies, diluted 1:1000 in PBS-0.3% Triton and 1% NGS or 1% NDkS, as follows: goat anti-mouse (Alexa Fluor 488, ThermoFisher Scientific, Cat#A-11001, RRID: AB_2534069), donkey anti-goat (Alexa Fluor 546, ThermoFisher Scientific, Cat#A-11056, RRID: AB_142628), goat polyclonal anti-mouse (Alexa Fluor 488, ThermoFischer Scientific, Cat#A-11001, RRID: AB_2534069), goat polyclonal anti-mouse (Alexa Fluor 546, ThermoFischer Scientific, Cat#A-11003, RRID: AB_2534071) goat polyclonal anti-rabbit (Alexa Fluor 488, ThermoFischer Scientific, Cat#A-11008, RRID: AB_143165), goat polyclonal anti-rabbit (Alexa Fluor 647, ThermoFischer Scientific, Cat#A-21244, RRID: AB_2535812). Nuclei were counterstained with DAPI 1:1000, diluted in PBS. Slides were then coverslipped with Fluoromount Aqueous Mounting Medium (Sigma-Aldrich, Cat#F4680). Digital images were acquired using Leica TCS SP8 confocal microscope.

### Immunohistochemistry

Spinal cord sections were washed in PBS, then blocked with 3% H_2_O_2_ in PBS for 15 min at RT. Slides were washed three times in PBS, then incubated for 1 h at RT with the following primary antibodies: mouse monoclonal anti-glial fibrillary acidic protein (Gfap) antibody (Santa Cruz Biotechnology, Cat#610,566, RRID: AB_397916, 1:100) and goat anti-AIF/Iba1 antibody (Novus Biologicals, Cat#NB100-1028, RRID: AB_521594, 1:100). Afterward, samples were washed in PBS-0.3% Triton, then incubated with biotinylated secondary antibody, diluted in PBS containing 1% bovine serum albumin (Horse Anti-Mouse/Rabbit/Goat IgG Antibody (H + L), Cat#BA-1300, RRID: AB_2336188, 1:200). After a 5 min wash, slides were incubated with VECTASTAIN Elite ABC-HRP Reagent (Vector Laboratories, Cat#PK-7100) for 30 min, at RT. Samples were then washed three times in PBS, then exposed to a solution of 1% DAB, 0.3% H_2_O_2_ in PBS until a brown coloration appeared. Nuclei were counterstained with Mayer’s Hematoxylin Solution (Sigma-Aldrich, Cat#MHS32). Sections were subsequently dehydrated using a series of increasing ethanol concentrations (50%, 70%, 95%, and 100%) and then cleared with xylene and coverslipped with Eukitt (Bio Optica, Cat#09–00250). Digital images were acquired using the Nexcope NIB600 biological microscope.

### Quantifications and statistical considerations

For neuron-specific TUJ1 analysis, neurite length was measured by using ImageJ software (Version 2.9.0 for Mac) and expressed as μm.

For immunohistochemical quantifications, the number of Gfap/Iba1 positive cells was calculated per unit area in each lamina of the ipsi-lateral dorsal horn of the spinal cord. The number of NeuN/Cleaved Caspase 3 (Cl Casp3), Gfap/Cl Casp3, and Iba1/Cl Casp3 double-positive cells was quantified by counting the number of double-positive cells per mm^2^ of randomized regions of interest (ROI) [34]. The fluorescence intensity for Gfap, Iba1, and Cx43 analyses was quantified using ImageJ software by calculating the mean fluorescence intensity (MFI) per area of randomized ROIs deriving from n = 4 ipsi-lateral dorsal horns of lumbar spinal cords of n = 4 rats per group.

All statistical analyses were performed using GraphPad Prism (Version 9.5.0 for Mac). The data were assessed for normality distribution by using the Shapiro–Wilk test, followed by an evaluation for homogeneity of variance. Data sets that passed both tests were analyzed by using One-way analysis of variance (ANOVA), and for comparisons between groups, Holm–Sidak’s multiple post-hoc test was applied. Statistical analyses of behavioral assessment of mechanical allodynia were performed using a two-way ANOVA repeated measure and Holm-Sidak’s multiple comparisons test. Unless otherwise specified, data are expressed as mean ± standard deviation (SD). For all statistical tests, p values < 0.05 were considered statistically significant.

## Results

### σ1R-antagonist resolves dorsal root ganglion neurons excitotoxic damage

**Fig. 1:**
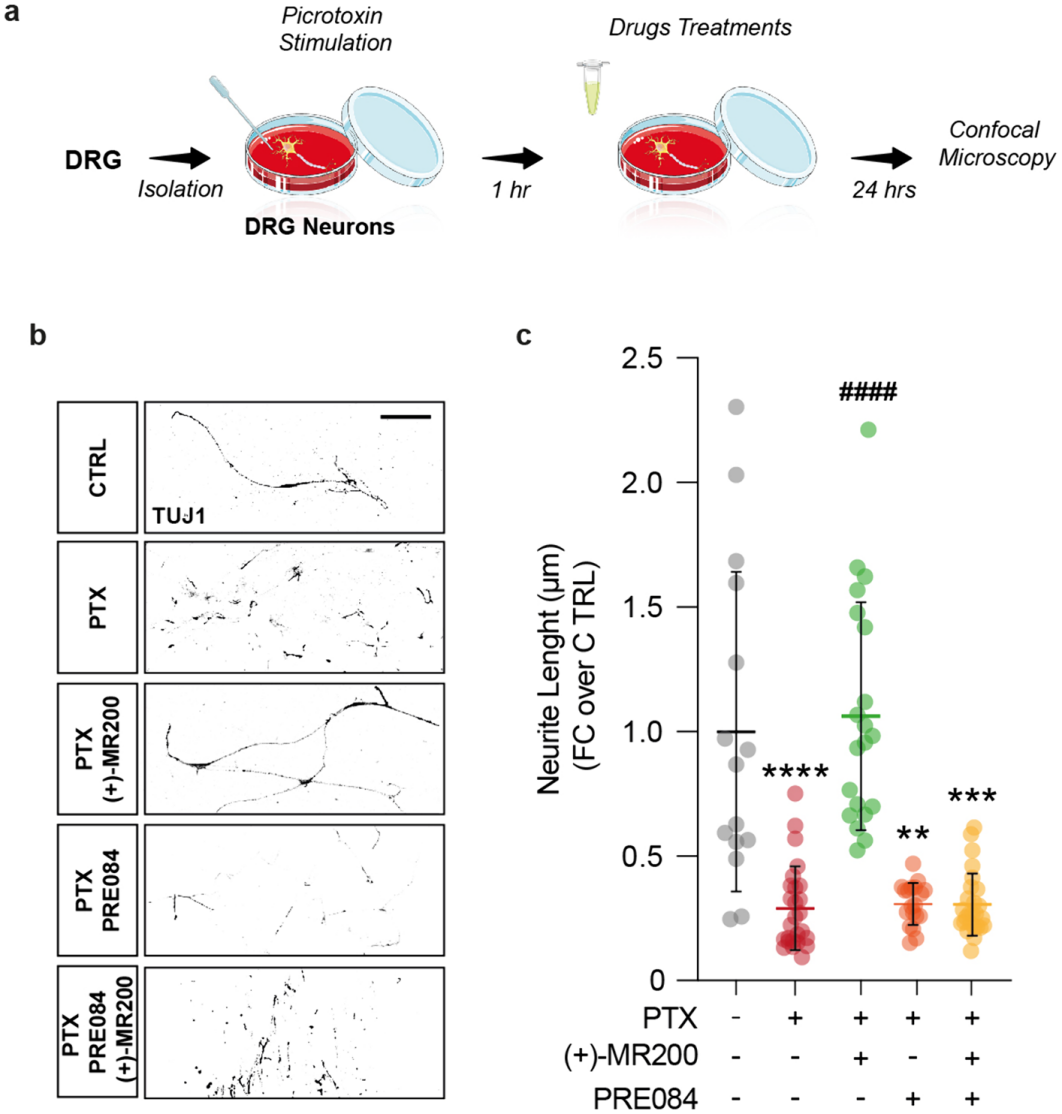
σ1R antagonist (+)-MR200 resolves PTX-induced excitotoxic damage upon DRG-derived neurons. **a** Experimental design of DRG-derived neurons cultures and treatments. **b** Representative pictures and **c** quantification of neurite length of TUJ1-positive DRG neurons. Data are shown as scattered dot plot and mean FC ± SD of n ≥ 15 neurites per group. *CTRL* control; *DRG* dorsal root ganglion; *FC* fold change; *PTX* picrotoxin; *SD* standard deviation. ****p-value < 0.0001, *** p-value < 0.001, **p-value < 0.01 versus CTRL, ^####^p-value < 0.0001 versus PTX. Scale bar 25 μm

In order to explore the potential of σ1R antagonism as a neuroprotective agent and exploitable target for neuropathic pain management, we decide to prior test our σ1R antagonist (+)-MR200, in an in vitro experimental model of excitotoxicity-induced inflammation. As such, we used primary DRG-derived neurons, exposed to 1 h stimulation with PTX, to induce excitotoxic damage. Subsequently, media was replaced with fresh medium added with 1 μM concentration of either (+)-MR200, PRE084 a well-established σ1R-agonist, or both (Fig. 1a). The effects of PTX-induced excitotoxicity on DRG neurons were analyzed revealing an important reduction of neurite length, of about 3.4 folds as compared to control (0.29 ± 0.17 PTX vs. 1.09 ± 0.64 CTRL; Fig. 1b, c). On the contrary, (+)-MR200 demonstrated the capability to counteract these neurodegenerative phenomena by exerting a protective effect on the overall sprouting of DRG neurons, leading to a significant increase in neurite length as compared to PTX-stimulated untreated cells (1.06 ± 0.45 (+)-MR200 vs. 0.29 ± 0.17 PTX; Fig. 1b, c). Co-administration of (+)-MR200 with the σ1R agonist PRE084, resulted in a reversal of the neuroprotective effects, leading to a significant reduction in both the overall sprouting of DRG neurons and neurite length compared to PTX-stimulated untreated cells (0.31 ± 0.13 PRE084 (+)-MR200 vs. 1.00 ± 0.6 CTRL; Fig. 1b, c). Notably, treatment with PRE084 alone did not exhibit a substantial impact on these neurodegenerative phenomena (0.31 ± 0.08 CCI PRE084; Fig. 1b, c). These findings demonstrated that (+)-MR200-mediated protective effects on DRG-derived neurons involve its action as a σ1R antagonist.

### (+)-MR200-mediate analgesic and anti-allodynic effects on CCI model of neuropathic pain

**Fig. 2:**
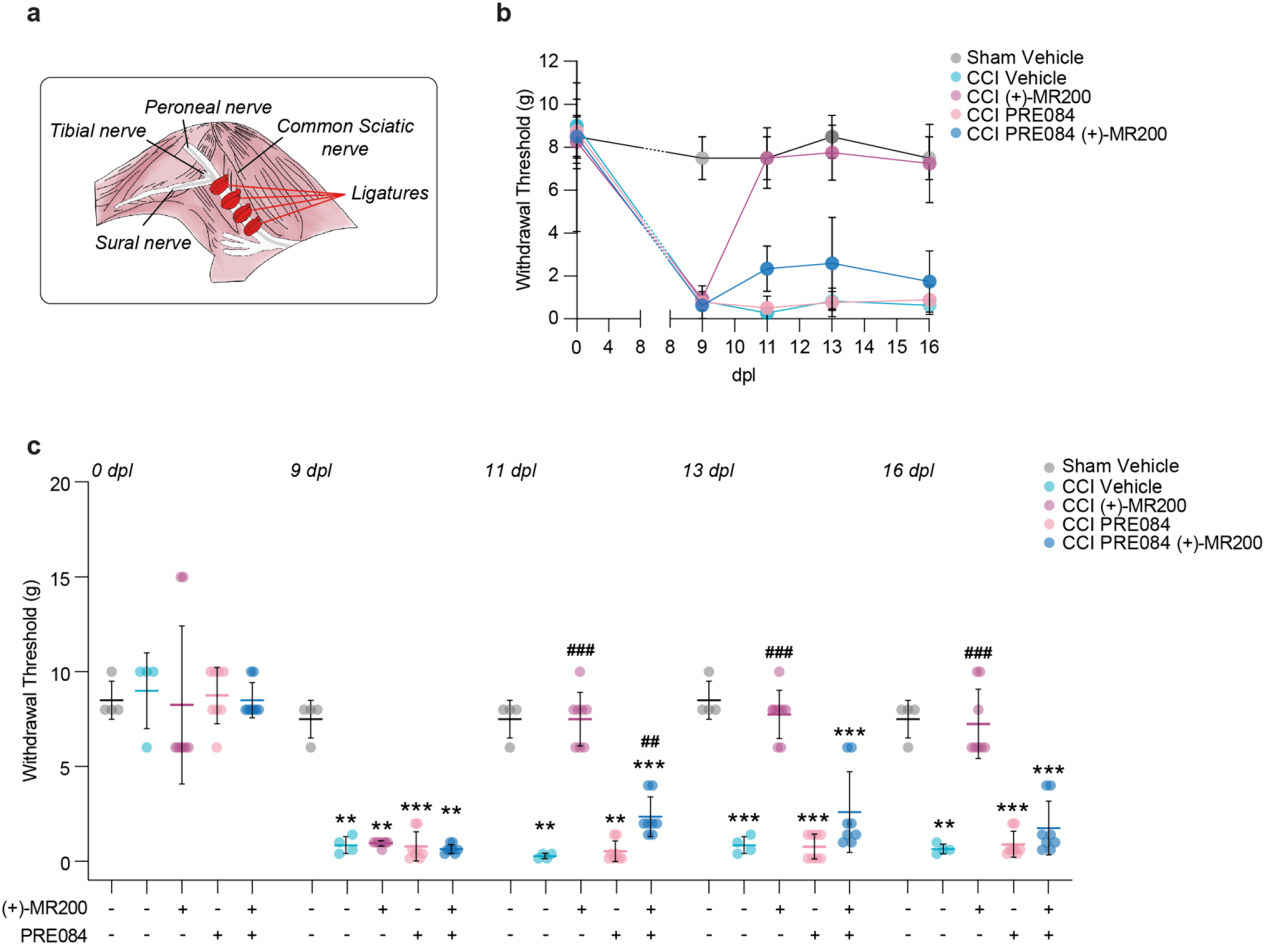
Effect of (+)-MR200 on withdrawal thresholds in response to von Frey stimulation in neuropathic rats. **a** Experimental paradigm of surgical procedure. **b**, **c**) Paw withdrawal thresholds on Sham vehicle, CCI vehicle, CCI (+)-MR200, CCI PRE084, CCI PRE084 (+)-MR200 at 0, 9, 11, 13 and 16 dpl. Data are shown as scattered dot plot and mean ± SD of n ≥ 4 animals per group. CCI, chronic constriction injury; dpl, days post ligatures, *SD* standard deviation. ***p-value < 0.001, **p-value < 0.01 versus Sham vehicle, ^###^p-value < 0.001 versus CCI vehicle

To investigate the therapeutic potential of (+)-MR200 compound during neuropathic pain we employed the well-established CCI model in male Sprague–Dawley rats. Briefly, four ligatures were applied around the left common sciatic nerve, proximal to the trifurcation, inducing neuropathic changes affecting nerve function and sensory perception (Fig. 2a).

Neuropathic pain establishment was assessed by evaluating mechanical allodynia, one of the prominent symptoms of neuropathy, using von Frey filaments. Baseline mechanical threshold was assessed before surgical procedures (i.e. 0 dpl). Notably, no significant differences were observed among the experimental groups, with consistent baseline thresholds recorded as follow: 8.5 ± 1.0 Sham Vehicle, 9.0 ± 2.0 CCI vehicle, 8.3 ± 4.2 CCI (+)-MR200, 8.8 ± 1.5 CCI PRE084, 8.5 ± 0.9 CCI PRE084 (+)-MR200 (Fig. 2b, c). By the 9 dpl, our data revealed a marked reduction in withdrawal thresholds for all treated group, in contrast to the sham-operated rats, which consistently maintained high thresholds (0.9 ± 0.4 CCI vehicle, 1.0 ± 0.1 CCI (+)-MR200, 0.8 ± 0.8 CCI PRE084 and 0.7 ± 0.2 CCI PRE084 (+)-MR200 vs. 7.5 ± 1.0 Sham vehicle, Fig. 2b, c).

CCI vehicle group showed a decrease in mechanical threshold that persisted up to 16 dpl (0.3 ± 0.2 CCI vehicle 11 dpl vs. 7.5 ± 1.0 Sham vehicle 11 dpl; 0.9 ± 0.4 CCI vehicle 13 dpl vs. 8.5 ± 1.0 Sham vehicle 13 dpl and 0.7 ± 0.3 CCI vehicle 16 dpl vs. 7.5 ± 1.0 Sham vehicle 16 dpl) (Fig. 2b, c). Pre-treatment with agonist alone, PRE084, did not affect paw withdrawal threshold as compared to CCI vehicle at 9 dpl (0.8 ± 0.8 CCI PRE084 vs. 0.9 ± 0.4 CCI vehicle), 11 dpl (0.5 ± 0.5 CCI PRE084 vs. 0.3 ± 0.1 CCI vehicle), 13 dpl (0.8 ± 0.7 CCI PRE084 vs. 0.9 ± 0.4 CCI vehicle) and 16 dpl (0.9 ± 0.7 CCI PRE084 vs. 0.7 ± 0.3 CCI vehicle; Fig. 2b, c). However, daily administration of (+)-MR200, resulted in an important recovery and improvement in mechanical allodynia, comparable to sham-control at 11 dpl (7.5 ± 1.4 CCI (+)-MR200), 13 dpl (7.8 ± 1.3 CCI (+)-MR200) and 16 dpl (7.3 ± 1.8 CCI (+)-MR200; Fig. 2b, c). Notably, this effect did not occur when (+)-MR200 was co-injected with PRE084. Indeed, our findings revealed a significant decrease in paw withdrawal threshold among the group receiving co-treatment compared to the sham group at the 9 dpl (0.7 ± 0.2 CCI PRE084 (+)-MR200) 11 dpl (2.4 ± 1.1 CCI PRE084 (+)-MR200), 13 dpl (2.6 ± 2.1 CCI PRE084 (+)-MR200) and 16 dpl (1.8 ± 1.4 CCI PRE084 (+)-MR200; Fig. 2b, c). Therefore, we concluded that the (+)-MR200 anti-allodynic effect is associated with its ability to act as an antagonist on the σ1R.

### σ1R targeting reduces spinal astrogliosis in neuropathic animals

**Fig. 3:**
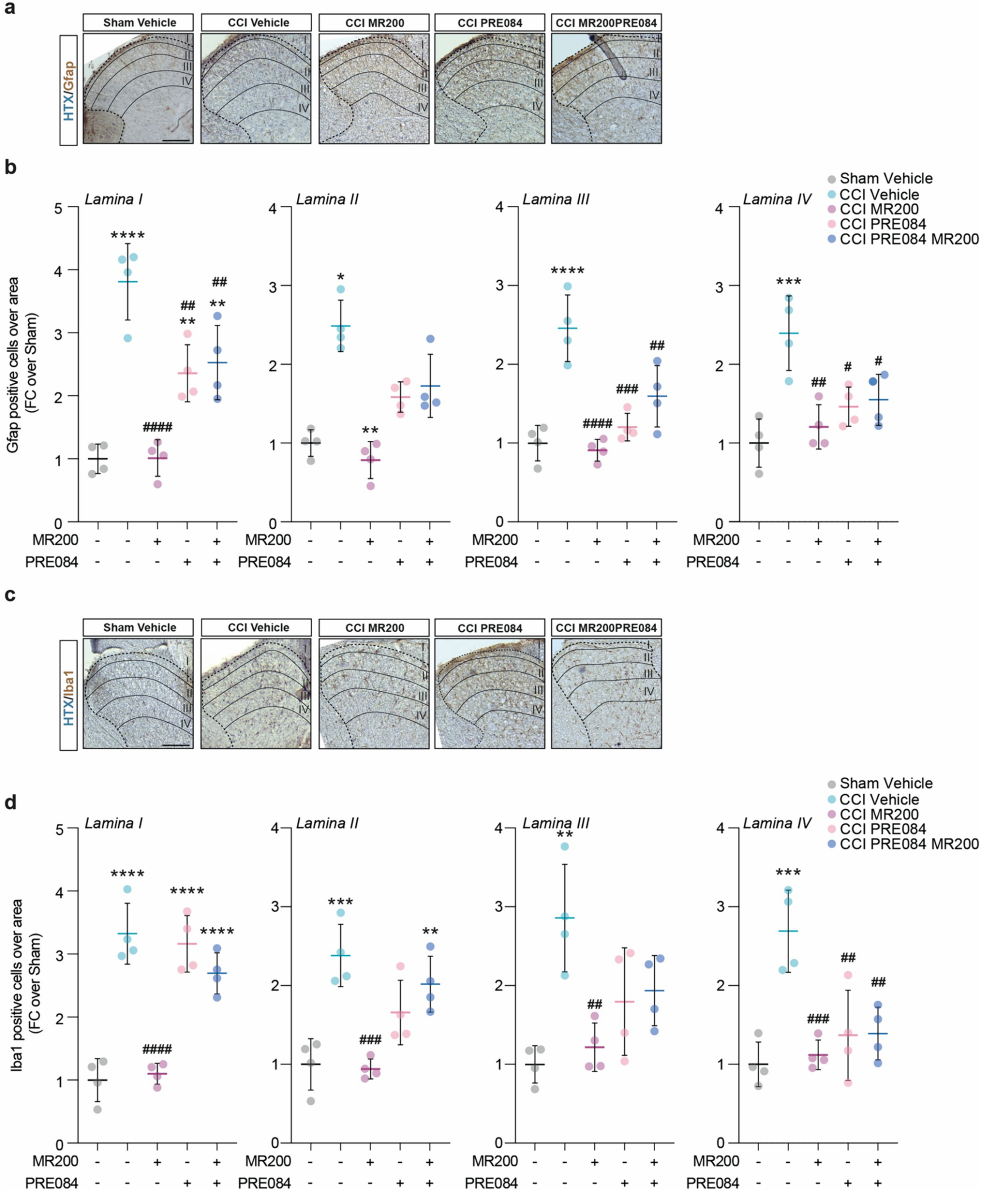
(+)-MR200 mitigates reactive astrogliosis at spinal cord level. **a** Representative pictures of lumbar sections of spinal cord showing Gfap-positive cells on laminae I-IV; **b** Quantification of Gfap-positive cells per laminae area; **c** Representative pictures of lumbar sections of spinal cord showing Iba1-positive cells on laminae I-IV; **d** Quantification of Iba1-positive cells per laminae area. Data are shown as scattered dot plot and mean FC ± SD of n = 4 sections per group. *CCI* chronic constriction injury; *FC* fold change; *SD* standard deviation. ****p-value < 0.0001, *** p-value < 0.001, **p-value < 0.01 versus Sham vehicle, ^####^p-value < 0.0001, ^###^p-value < 0.001, ^##^p-value < 0.01, ^#^p-value < 0.05 versus CCI vehicle. Scale bar 100 μm

We then proceed to study the link between beneficial effects on behavioral threshold and the underlying mechanism at spinal cord level. Through immunohistochemical analyses, our data reveal an increase in the proportion of astrocytes (i.e., Gfap positive cells) in ipsilateral laminae I-IV of the lumbar region of spinal cord biopsies of CCI vehicle rats as compared to sham controls (3.81 ± 0.61 lamina I CCI vehicle, 2.45 ± 0.33 lamina II CCI vehicle, 2.46 ± 0.42 lamina III CCI vehicle, 2.40 ± 0.47 lamina IV CCI vehicle, FC over sham vehicle Fig. 3a, b).

We also observed a similar increase in the proportion of microglia cells in CCI-operated animals (i.e., Iba1 positive cells) in lamina I (3.32 ± 0.49 CCI vehicle), lamina II (2.38 ± 0.40 CCI vehicle), lamina III (2.86 ± 0.68 CCI vehicle) and lamina IV (2.69 ± 0.53 CCI vehicle) as compared to sham vehicle rats (Fig. 3c, d). Such an effect was abolished by (+)-MR200 treatment which reestablished the levels of glial cells in the ipsilateral laminae I-IV near to sham levels. In particular, we observed a significant reduction in the proportion of Gfap-expressing cells (1.01 ± 0.29 lamina I, 0.78 ± 0.23 lamina II, 0.91 ± 0.14 lamina III, 1.21 ± 0.28 lamina IV CCI (+)-MR200, FC over sham; Fig. 3a, b) and Iba1-expressing cells (1.10 ± 0.17 lamina I, 0.94 ± 0.13 lamina II, 1.22 ± 0.31 lamina III, 1.12 ± 0.19 lamina IV CCI (+)-MR200, FC over Sham vehicle; Fig. 3c, d) as compared to CCI vehicle animals. Single treatment with PRE084 did not affect the proportion Gfap + cells in laminae I-IV (2.36 ± 0.45 lamina I, 1.58 ± 1.19 lamina II, 1.20 ± 0.18 lamina III, 1.46 ± 0.25 lamina IV CCI PRE084, FC over Sham vehicle; Fig. 3a, b) and Iba1 + cells in laminae I-IV (3.16 ± 0.45 lamina I, 1.66 ± 0.41 lamina II, 1.80 ± 0.68 lamina III, 1.37 ± 0.57 lamina IV CCI PRE084, FC over Sham vehicle; Fig. 3c, d), maintaining levels similar to CCI vehicle group, significantly higher compared to sham control (Fig. 3a–d). Of note, cotreatment with (+)-MR200 and PRE084 was able to reverse (+)-MR200-mediated effect, showing similar levels of Gfap + cells in laminae I-IV (2.53 ± 0.59 lamina I, 1.73 ± 0.40 lamina II, 1.59 ± 0.39 lamina III, 1.55 ± 0.32 lamina IV CCI PRE084 (+)-MR200, FC over Sham vehicle; Fig. 3a, b) and Iba1 + cells in laminae I-IV (2.69 ± 0.32 lamina I, 2.02 ± 0.36 lamina II, 1.93 ± 0.45 lamina III, 1.39 ± 0.33 lamina IV CCI PRE084 (+)-MR200, FC over Sham vehicle, Fig. 3c, d) as compared to CCI untreated group (Fig. 3a–d). Taken together, these data indicate that (+)-MR200 exerts its beneficial effect acting as a σ1R antagonist, influencing spinal cord dorsal horn resident cell population and promoting an anti-inflammatory phenotype.

### (+)-MR200 counteracts pro-apoptotic profile of spinal cord cell population induced by CCI

**Fig. 4:**
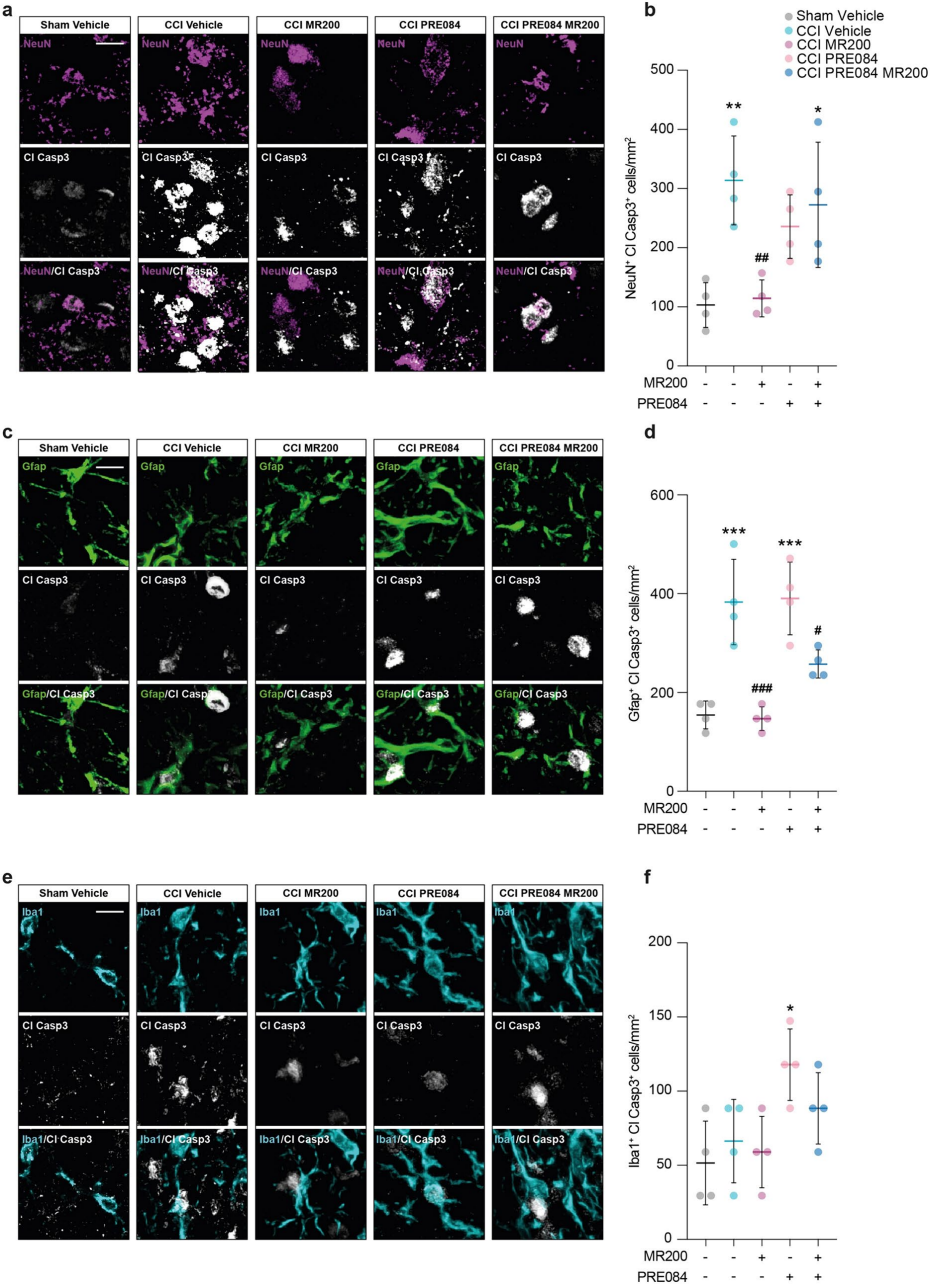
**(+)-**MR200 rescues pro-apoptotic phenotype following CCI. **a** Representative confocal microscopy images of NeuN + Cl Casp3 + cells in ipsi-lateral dorsal horn. **b** Quantification of NeuN + Cl Casp3 + cells per mm^2^. **c** Representative confocal microscopy images of Gfap + Cl Casp3 + cells in ipsi-lateral dorsal horn. **d** Quantification of Gfap + Cl Casp3 + cells per mm^2^. **e** Representative confocal microscopy images of Iba1 + Cl Casp3 + cells in ipsi-lateral dorsal horn. **f** Quantification of Iba1 + Cl Casp3 + cells per mm^2^. Data are shown as scattered dot plot and mean FC ± SD of n = 4 sections per group. *CCI* chronic constriction injury; *Cl* Casp3, cleaved caspase 3; *FC* fold change; *SD* standard deviation. *** p-value < 0.001, **p-value < 0.01, *p-value < 0.05 versus Sham vehicle, ^###^p-value < 0.001, ^##^p-value < 0.01, ^#^p-value < 0.05 versus CCI vehicle. Scale bar 10 μm

We then moved to understand the mechanistic contributions of σ1R targeting at spinal level, investigating the pathophysiological changes in cellular state as a result of reactive gliosis. We stained spinal cord sections for Cl Casp3 as a marker of cell death and apoptotic signaling to assess the cellular state of neurons (i.e., NeuN positive cells), astrocytes (i.e., Gfap positive cells) and microglia (i.e., Iba1 positive cells) in response to nerve injury and under (+)-MR200 treatment. Our data reveal a robust increase of NeuN/Cl Casp 3 double positive cells in CCI vehicle group as compared to sham-operated rats (314 ± 75.0 CCI vehicle vs. 103 ± 38.0 Sham vehicle; Fig. 4a, b).

Such an increase was significantly attenuated by the (+)-MR200-mediated effect on the σ1R, as compared to CCI vehicle group (114 ± 31.2 CCI (+)-MR200 vs. 314 ± 75.0 CCI vehicle; Fig. 4a, b). Of note, treatment with the agonist PRE084 or combination of PRE084 and (+)-MR200 did not exert neuroprotective effects, with an increase of NeuN + Cl Casp3 + cells as compared to Sham vehicle group (236 ± 53.8 CCI PRE084 and 272 ± 106 CCI PRE084 (+)-MR200 vs. 103 ± 38.0 Sham vehicle; Fig. 4a, b).

We then moved to analyze the proportion of Gfap/Cl Casp3 double positive cells, finding a significant increase in CCI-untreated animals as compared to sham-operated rats (383 ± 86.7 CCI vehicle vs. 155 ± 28.2 Sham vehicle; Fig. 4c, d). On the contrary, (+)-MR200 effectively attenuates this condition, resulting in a decrease in the proportion of Gfap + Cl Casp3 + as compared to CCI vehicle animals (147 ± 24.1 CCI (+)-MR200 vs. 383 ± 86.7 CCI vehicle; Fig. 4c, d). Furthermore, our evidence showed that neither PRE084 alone nor in combination with (+)-MR200 was effective in reducing the increased proportion of Gfap + Cl Casp3 + cells observed after CCI compared to the sham-control group (390 ± 73.6 CCI PRE084 and 250 ± 28.2 CCI PRE084 (+)-MR200 vs 155 ± 28.2 Sham vehicle; Fig. 4c, d). Notably, a slight modulation was observed in the proportion of Iba1 + cells, also expressing Cl Casp3 in each condition (66.3 ± 28.2 CCI vehicle, 58.0 ± 24.1 CCI (+)-MR200, 118 ± 24.1 CCI PRE084, 88.4 ± 24.1 CCI PRE084 (+)-MR200 vs. 51.6 ± 28.2 Sham vehicle, Fig. 4e, f). Collectively, these findings suggest that (+)-MR200 is able to resolve the CCI-induced pro-apoptotic signaling, effectively ameliorating cellular survival and responses.

### σ1R antagonism influences cell-to-cell communication by regulating Cx43 trafficking

**Fig. 5:**
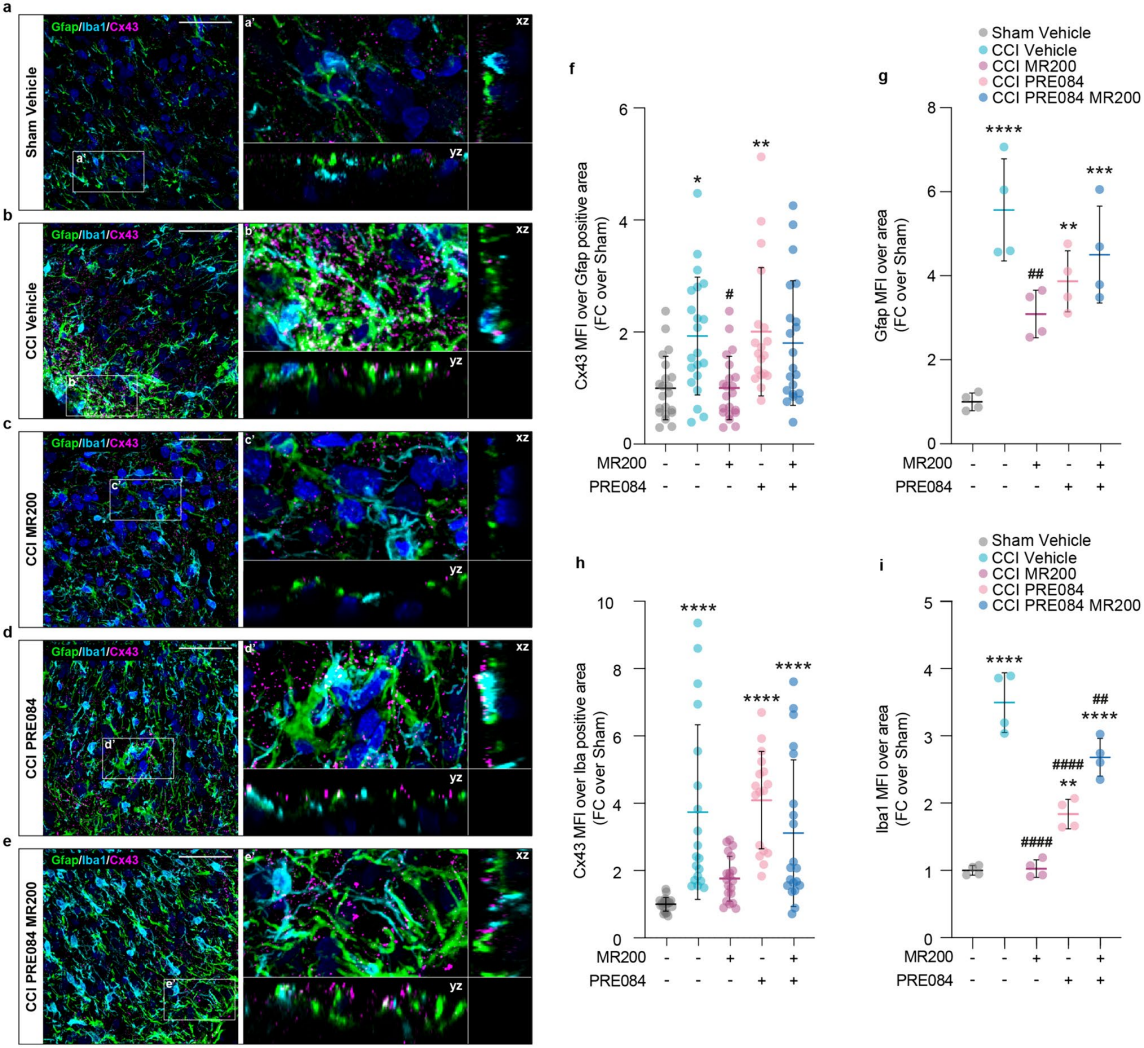
**(+)-**MR200 treatment influences Cx43-mediated cell-to-cell communication. **a**–**e** Confocal microscopy images of Gfap-positive astrocytes and Iba1-positive microglia expressing Cx43 in ipsilateral dorsal horn of Sham vehicle (**a**), CCI vehicle (**b**), CCI (+)-MR200 (**c**), CCI PRE084 (**d**), CCI PRE084 (+)-MR200 (**e**). a’–e’) Magnification and orthogonal projections showing Cx43 localization between astrocytes and microglia. **f** Quantification of Cx43 MFI over Gfap positive area and h) quantification of Cx43 MFI over Iba1 positive area. Data are shown as scattered dot plot and mean FC ± SD of n > 16 positive cells per group. g) Quantification of Gfap MFI over total area and **i** quantification of Iba1 MFI over total area. Data are shown as scattered dot plot and mean FC ± SD of n = 4 samples per group. CCI, chronic constriction injury; FC, fold change; MFI: mean fluorescence intensity; SD, standard deviation. ****p-value < 0.0001, **p-value < 0.01, *p-value < 0.05 versus Sham vehicle, ^####^p-value < 0.0001, ^##^p-value < 0.01, ^#^p-value < 0.05 versus CCI vehicle. Scale bar 50 μm

Based on our previous findings, demonstrating the impact of σ1R antagonism on cell-to-cell communication and Cx43 modulation [15], we further explored spinal cord dynamics, evaluating the contribution of GJs-mediated intercellular communication in regulating the cellular response to CCI. Using immunofluorescence-based analyses, we found an increase in the Cx43-Gfap colocalization profile in CCI vehicle group as compared to sham controls, which was reversed by (+)-MR200 treatment (1.93 ± 1.05 CCI vehicle and 1.00 ± 0.57 CCI (+)-MR200, FC over Sham vehicle; Fig. 5a–e, f). In contrast, treatment with PRE084 did not affect the increased Cx43-Gfap colocalization profile induced by CCI, and the co-administration counteracted the effects exerted by (+)-MR200 (2.00 ± 1.14 CCI PRE084 and 1.80 ± 1.11 CCI PRE084 (+)-MR200, FC over Sham vehicle; Fig. 5a–e, f). Along with these findings, we also notice a robust increase of about 4.5 folds of Gfap MFI in the ipsi-lateral dorsal horn of the spinal cord in CCI vehicle group in relation to sham controls (5.57 ± 1.94 CCI vehicle, FC over Sham vehicle; Fig. 5a–e, g).

Such an increase may suggest an upregulation or activation of glial cells in response to injury. This effect was completely abolished by (+)-MR200 treatment as compared to CCI vehicle, leading to near normal levels (3.09 ± 0.56 CCI (+)-MR200 vs. 5.57 ± 1.94 CCI vehicle, FC over sham; Fig. 5a–e, g). Furthermore, our evidence indicates that CCI PRE084- and CCI PRE084 (+)-MR200-treated rats showed similar levels of Gfap MFI in relation to CCI vehicle group, significantly higher as compared to sham vehicle rats (3.87 ± 0.80 CCI PRE084 and 4.51 ± 1.32 CCI PRE084 (+)-MR200, FC over Sham vehicle; Fig. 5a–e, g). A strong increase in Cx43 MFI was also observed over Iba1-positive area in CCI vehicle group as compared to sham vehicle group (3.74 ± 2.59 CCI vehicle, FC over Sham vehicle; Fig. 5a–e, h), significantly diminished by (+)-MR200 treatment (1.76 ± 0.66 CCI (+)-MR200, FC over Sham vehicle; Fig. 5a–e, h).

Similar differences were also detected by quantifying Iba1 MFI the ipsi-lateral dorsal horn of the spinal cord in CCI vehicle group in relation to sham controls (3.50 ± 1.60 CCI vehicle, FC over Sham vehicle; Fig. 5a–e, i), significantly reduced by (+)-MR200 treatment (1.04 ± 0.60 CCI (+)-MR200; Fig. 5a–e, i). Importantly, PRE084 alone or in cotreatment with (+)-MR200, were able to reverse (+)-MR200-mediated effects showing similar levels of Cx43/Iba1 colocalization profile (4.09 ± 1.45 CCI PRE084, 3.11 ± 2.18 CCI PRE084 (+)-MR200) and Iba1 MFI (3.78 ± 0.80 CCI PRE084, 4.51 ± 1.32 CCI PRE084 (+)-MR200) as compared to the CCI vehicle-treated group and significantly higher as compared to the sham-operated controls (Fig. 5a–e, h). Taken together these findings suggest that CCI induces an overstimulation of astrocytes and microglial cells, as evidenced by the increased MFI intensity of Gfap and Iba1 at the spinal level, which was coupled with an up-regulation of Cx43 at the membrane interface of both cell populations. Thus, the neuroprotective and anti-allodynic effect of (+)-MR200, mediated by σ1R antagonism, correlates with reduced Cx43 expression and consequently reduced intercellular exchanges.

## Discussion

Our understanding of neuropathic pain has evolved significantly over time, incorporating insights from neurobiology, immunology and pharmacology. The chronicization process is a comprehensive phenomenon involving both neuronal and glial cells, encompassing a combination of peripheral and central sensitization mechanisms [35–38]. Peripheral sensitization entails changes in the excitability of nociceptive neurons, resulting in heightened responses to normally non-painful stimuli. In contrast, central sensitization involves alterations in the function and plasticity of neurons in the spinal cord and brain, amplifying and prolonging pain signals [39–41]. The molecular and cellular processes underpinning sensitization affect voltage-sensitive ion channels, including sodium, calcium and potassium channels, along with nociceptor sensitization and abnormal ectopic excitability of afferent neurons [42–46]. This reshaping of CNS and PNS molecular players can be considered a critical hallmark of neuropathic pain.

In this context, it has been reported that σ1Rs play opposite roles in CNS, being implicated in both neurotoxicity [47, 48] and neuroprotective functions [49–52]. Their involvement extends to the regulation of crucial aspects such as calcium homeostasis, excitotoxicity, oxidative stress, as well as endoplasmic reticulum and mitochondrial stress [53–55]. Notably, the observed preventive impact of σ1R antagonism on peripheral neuropathy, demonstrated through genetic inactivation (i.e. σ1R knockout mice) and pharmacological blockade, has proven effective in counteracting paclitaxel-induced sensory nerve mitochondrial abnormalities and associated pain hypersensitivity [56]. However, conflicting findings in research suggest that the σ1R agonist SA4503, rather than the σ1R antagonist NE-100, exhibits antinociceptive effects against chemotherapy-induced neuropathic pain in rats [57]. These pieces of evidence prompted us to assess in vitro the potential effect of (+)-MR200, as a σ1R antagonist, and PRE084, as a σ1R agonists, on PTX-induced toxicity on neurons. Our data showed that (+)-MR200 prevented neurite damage induced by discharge, whereas single treatment with the σ1R agonist or cotreatment with antagonists did not preserve neurite length in isolated neurons. These findings align with previously reports demonstrating the neuroprotective effects of σ1R antagonists [51]. Importantly, we also confirmed in vitro that the σ1R agonist PRE084 failed to induce a neuroprotective effect on PTX-stimulated neurons, as reported by Luedtke and coworkers in a different model using the σ1R agonist (+)-pentazocine [49].

In an effort to test the effects of σ1R modulation in vivo, we studied the effects of (+)-MR200 and PRE084 in a model of neuropathic pain. While (+)-MR200 reduced typical behavioral features of CCI rats, neither the single σ1R agonist administration nor the cotreatment between (+)-MR200 and PRE084 exerted any significant beneficial effects on CCI-induced mechanical allodynia. This supports the hypothesis of a protective role of σ1R antagonists in chronic neuropathic pain. Further analysis on the proportion of Cl Casp3 positive neurons pointed towards the same conclusion. However, some considerations should be taken into account. On the one hand, we observed a significant reduction in the proportion of suffering neurons in (+)-MR200-treated rats, and this effect was fully reverted by cotreatment with PRE084. On the other hand, our analysis showed that treatment with PRE084 alone, even if not inducing any significant behavioral amelioration, were coupled with a slight, although not significant, reduction in the proportion of Cl Casp3 positive neurons.

Advances in neuroimaging techniques and molecular biology have played a crucial role in shaping our understanding of CNS pathophysiology, emphasizing the significance of microglia and astrocytes in sustaining the chronicization of neuropathic pain [58, 59]. Communication between these glial cells regulates homeostasis in the CNS, influencing various physio-pathological functions, including synaptic transmission and inflammatory processes [60]. Reactive activation of glial cells has been reported as a leading factor in fostering neuroinflammation and subsequent degenerative stimuli, linked with microenvironmental conditioning via the release of pro-inflammatory mediators [61].

Our data on the astrogliosis and microgliosis in the sensory laminae suggested a significant increase of reactive astrocytes and a similar magnitude of increase in reactive microglial cells in CCI rats, in accordance with previous evidence [12]. While (+)-MR200 treatment prevented reactive gliosis in laminae I-IV, the σ1R agonist PRE084 reverted (+)-MR200-induced effects in laminae I, II, and, at least partially, in the deepest laminae. This is important in terms of (+)-MR200 efficacy in reducing gliosis and neuronal suffering in laminae involved in the transmission of tactile and thermal sensory information (i.e. lamina III) and sensory information related to deep sensation and proprioception (i.e. lamina IV). Moreover, our data showed a slight modulation mediated by PRE084 in regulating gliosis at these sites. This apparent contradiction may be due to the dual effect of σ1R ligands. σ1R modulation participates in different interconnected neuroprotective mechanisms, from the regulation of neuronal excitability and calcium trafficking to the tuning of glial cells reactivity. The σ1R functions as a chaperone protein, thus without a traditional transduction system as for other receptors. The effects of σ1R ligands are highly context-dependent, varying with the specific cellular environment and pathological conditions. It is known that σ1R ligands can influence calcium signaling at the mitochondrial-endoplasmic reticulum membrane [62]. While the agonist enhances the transfer of calcium from the endoplasmic reticulum to mitochondria, which is protective under normal conditions, but can lead to mitochondrial calcium overload and cell death under pathological conditions. Conversely, antagonist may prevent this excessive calcium transfer, protecting mitochondria from stress but potentially leaving cells energetically deprived [63]. This means that agonist may induce stress responses that are beneficial in early stages of neuronal damage by enhancing cellular resilience. Antagonists, on the other hand, might inhibit excessive reactive responses, providing a protective effect in chronic condition. For instance, in vivo modulation of the σ1R by treatment with agonist ligands has been shown to regulate multiple aspects of astrocytes and microglial activity in various neuropathological conditions linked to neuronal death [64]. Interestingly, PRE084 treatment in spinal muscular atrophy mice attenuated reactive gliosis and restored the disrupted M1/M2 phenotype balance, promoting M2 polarization and an anti-inflammatory response in microglia [65]. Wang and colleagues showed that modulation of the σ1R by SA4504, considered as an agonist, reduced astroglia reactivity in LPS-activated astrocytes, in vitro [66]. This duality underscores the receptor’s role in maintaining a delicate balance of cellular processes.

Our data also support the effects of (+)-MR200 as a σ1R targeting drug in the CNS, given that PRE084 has been reported to be rapidly distributed to CNS, with similar concentrations in the brain, spinal cord, and plasma when administered i.p. [67].

Neuroinflammation is recognized as a significant contributor to neuropathic pain. Pro-inflammatory cytokines and chemokines released by reactive glial cells trigger plastic changes and increase neuronal discharge, exacerbating the underlying condition [68]. In our study we also observed an increase in Gfap and Iba1 MFI in CCI rats, indicating the heightened activation of these cells. Interestingly, (+)-MR200 treatment reduced the CCI-induced astrogliosis, restoring the phenotype of these cells close to that of sham controls. This effect may be related to the chaperone mechanism mediated by the σ1R activity. Roh and colleagues observed that treatment with BD-1047, a well-known σ1R antagonist, blocked the CCI-induced phosphorylation of NMDA receptor subunit 1, which is responsible for the excessive calcium influx and the subsequent downstream effects [69]. This reduction in calcium-mediated signaling may attenuate astrocyte activation and thereby mitigating reactive astrogliosis. A decrease in microgliosis in the spinal cord was also previously reported by using BD-1047 in the early stages of chronic pain in models of osteoarthritis in mice [70]. These findings were coupled with a reduction in pro-inflammatory cytokines levels, such as TNF or IL-1β [71]. In addition, the modulation of astrocytic activity by σ1R antagonists has been linked to the inhibition of nuclear factor (NF-kB) signaling, which contributes to neuroinflammation [62].

Targeting specific components of the neuroinflammatory cascade has shown promise in preclinical settings, opening avenues for the development of novel anti-inflammatory therapies. For this reason, we also assessed the effect of σ1R antagonist and agonist on glial cells by quantifying the proportion of Cl Casp3 positive astrocytes and microglia. This set of evidence showed the highest level of divergence between these modulators. We observed that (+)-MR200 abolished the proportion of Cl Casp3 astrocytes, while PRE084 did not induce any significant modulation on the astroglial phenotype at a central level. This evidence aligns with a previous report suggesting that σ1R knockout mice were characterized by an attenuated nociceptive response in preclinical models of central neuropathic pain [72]. This phenomenon has been linked to a significant reduction of pro-inflammatory mediators, such as TNF, IL-1β, IL-6, and NR2B-NMDA over activation [73], as well as a significant reduction in intercellular coupling [15].

Our data also revealed an unexpected increase in the proportion of microglial cells positive for Cl Casp3 in rats exposed to the σ1R agonist PRE084. While this phenomenon was not observed in control CCI rats, a slight increase of suffering microglial cells was found in PRE084 treated CCI rats, indicating pleiotropic effects on microglia that are worthy of further investigations. Here, we focused on the cellular mechanisms sustaining pain chronicization and further expand our study on the role of GJs and Cxs, crucial for intercellular communication, allowing the direct exchange of molecules and cross-cellular conditioning [14, 74]. Our results suggest a significant increase in Cx43 expression profile in the spinal cord of CCI animals. This upregulation paralleled the activation of astrocytes and microglia, suggesting a potential link between increased Cx43 levels and reactive gliosis in neuropathic conditions. Inhibition of GJs function has shown promise in reducing inflammatory and neuropathic pain, highlighting the potential translational impact of targeting Cx43. We previously reported evidence of an astrocyte-Cx43-microglia axis in sustaining a long-term maintenance of neuropathic pain, providing a litmus test for novel therapeutic intervention [75]. Our study provided evidence that the σ1R antagonist (+)-MR200 significantly reduced Cx43 expression levels on both astrocytes and microglial cells, and this effect was not observed when targeting σ1R with the agonist nor when rats were exposed to both drugs. The mechanism by which σ1R modulates Cx43 expression and function likely relates to its role as a signaling modulatory chaperone. In the CNS, σ1Rs are primarily located on the endoplasmic reticulum membrane. Upon activation, σ1Rs can translocate to the cell surface, where they interact with various proteins, including those forming GJs, potentially affecting their activity. This hypothesis is further corroborated by the spatial proximity between the 2 proteins observed in a model of spinal cord injury [76]. Consequently, Cx43 presents itself as a potential biomarker and therapeutic target for countering neuroinflammation and pain chronicization. While the molecular mechanisms underlying σ1R involvement in neuropathic pain remain an area of active investigation, this study suggests that they may exert their analgesic effects through interactions with glial cells and reducing intercellular exchanges mediated by Cx43-based channels.

## Conclusions

The evolving role of σ1Rs in managing neuropathic pain presents significant potential for the development of novel and effective therapies. Further research is essential to uncover the mechanisms that underlie σ1R-mediated pain relief, particularly in its interactions with glial cells and neuroinflammatory processes. Investigating the dynamic changes in σ1R expression and function during chronic pain progression will be crucial for optimizing therapeutic strategies. Additionally, exploring the potential synergistic effects of combining σ1R antagonists with other established pain medications may offer more comprehensive and enduring pain relief.

In conclusion, σ1Rs have emerged as promising targets for novel therapeutic strategies in the management neuropathic pain. Clarifying interactions with glial cells and neuroinflammation holds a significant potential for neuropathic pain relief, ultimately improving the quality of life for millions suffering from this debilitating condition. The exploration of σ1Rs provides a ray of optimism for the development of future effective and precisely targeted strategies for innovative approaches to pain management.

## Data Availability

The data supporting the findings of this study are available from the corresponding author upon reasonable request.

## Abbreviations

AIF1/Iba1: Allograft inflammatory factor 1
CCI: Chronic constriction injury
Cl Casp 3: Cleaved caspase 3
CNS: Central nervous system
Cx43: Connexin 43
Dpl: Days post-ligatures
DRG: Dorsal root ganglion
Gfap: Glial fibrillary acid protein
GJ: Gap junction
MFI: Mean fluorescence intensity
NeuN: Neuronal nuclear protein
PBS: Phosphate-buffered saline
PFA: Paraformaldehyde
PNS: Peripheral nervous system
PTX: Picrotoxin
σR: Sigma receptor
